# Measuring the physical and economic impact of filarial lymphoedema in Chikwawa district, Malawi: a case-control study

**DOI:** 10.1186/s40249-017-0241-2

**Published:** 2017-04-03

**Authors:** Michelle C. Stanton, Masato Yamauchi, Square Z. Mkwanda, Paul Ndhlovu, Dorothy Emmie Matipula, Charles Mackenzie, Louise A. Kelly-Hope

**Affiliations:** 1 Department of Parasitology, Liverpool School of Tropical Medicine, Liverpool, UK; 2 Ministry of Health, Lilongwe, Malawi; 3 Chikwawa District Health Office, Chikwawa, Malawi; 4 Chiradzulu District Health Office, Chiradzulu, Malawi; 5 Department of Pathobiology and Diagnostic Investigation, Michigan State University, East Lansing, USA

**Keywords:** Lymphatic filariasis, Lymphoedema, Morbidity, Malawi, GPS, Mobility, Quality of life

## Abstract

**Background:**

Lymphatic filariasis (LF) is one of the primary causes of lymphoedema in sub-Saharan Africa, and has a significant impact on the quality of life (QoL) of those affected. In this paper we assess the relative impact of lymphoedema on mobility and income in Chikwawa district, Malawi.

**Methods:**

A random sample of 31 people with lymphoedema and 31 matched controls completed a QoL questionnaire from which both an overall and a mobility-specific score were calculated. Two mobility tests were undertaken, namely the 10 m walking test [10MWT] and timed up and go [TUG] test, and a subset of 10 cases-control pairs wore GPS data loggers for 3 weeks to measure their mobility in a more natural setting. Retrospective economic data was collected from all 31 case-control pairs, and each participant undertaking the GPS activity recorded daily earnings and health expenditure throughout the observation period.

**Results:**

Cases had a significantly poorer overall QoL (cases = 32.2, controls = 6.0, *P* < 0.01) and mobility-specific (cases = 43.1, controls = 7.4, *P* < 0.01) scores in comparison to controls. Cases were also significantly slower (*P* < 0.01) at completing the timed mobility tests, e.g. mean 10MWT speed of 0.83 m/s in comparison to 1.10 m/s for controls. An inconsistent relationship was observed between mobility-specific QoL scores and the timed test results for cases (10MWT correlation = −0.06, 95% *CI* = (−0.41, 0.30)), indicating that their perceived disability differed from their measured disability, whereas the results were consistent for controls (10MWT correlation = −0.61, 95% *CI* = (−0.79, −0.34)). GPS summaries indicated that cases generally walk shorter distances at slower speeds than control, covering a smaller geographical area (median area by kernel smoothing: cases = 1.25 km^2^, controls = 2.10 km^2^, *P* = 0.16). Cases reported earning less than half that earned by controls per week (cases = $0.70, controls = $1.86, *P* = 0.064), with a smaller proportion of their earnings (16% vs 22%, *P* = 0.461) being spent on healthcare.

**Conclusions:**

Those affected by lymphoedema are at a clear disadvantage to their unaffected peers, experiencing a lower QoL as confirmed by both subjective and objective mobility measures, and lower income. This study also indicates that objective measures of mobility may be a useful supplement to self-assessed QoL questionnaires when assessing the future impact of lymphoedema management interventions.

**Electronic supplementary material:**

The online version of this article (doi:10.1186/s40249-017-0241-2) contains supplementary material, which is available to authorized users.

## Multilingual abstract

Please see Additional file 1 for translations of the abstract into five official working languages of the United Nations.

## Background

Lymphoedema, a chronic condition seen clinically as localised swelling commonly in the legs or arms, is one of the classical clinical symptoms of lymphatic filariasis (LF), with approximately 17 million being affected globally [1]. In addition to the swelling, which can cause discomfort and problems with mobility [2], those with filarial lymphoedema are frequently affected by repeated episodes of acute dermatolymphangioadenitis (ADLAs), referred to as acute attacks. These attacks, which include pain, fever and increased swelling of affected areas can be extremely debilitating and are further associated with the progression of the severity of the lymphoedema. Together, lymphoedema and acute attacks have a significant social [3], psychological [4, 5] and economic [6, 7] impact on the affected individual. As such, the Global Programme to Eliminate Lymphatic Filariasis (GPELF) recognises that in order to eliminate LF as a public health problem, these conditions need to be effectively managed [8, 9]. Lymphoedema management primarily consists of simple hygiene measures such as frequent washing and exercise, with there being strong evidence that these measures lower the number of acute attacks, improve self-assessed quality of life, and decrease swelling [10].

In Malawi, the national LF elimination programme has been very successful in reducing the transmission of the disease through annual mass drug administration with ivermectin and albendazole [11, 12], and is now in a position to focus on reducing the associated morbidity burden. Recent studies in Chikwawa district, Southern Region have focused on quantifying the number of people affected by the clinical symptoms of LF [13, 14], and measuring the self-assessed quality of life of those affected [15]. These studies involved classifying the severity of lymphoedema into three categories (mild, moderate, severe), corresponding to Dreyer stages 1–2, 3–5 and 5–7 respectively, according to the degree of swelling and the presence of skin folds [13, 16]. Whilst Martindale et al. [15] provided a valuable insight into the burden placed on those affected by their condition, their study was limited to subjective measures of quality of life/disability, and no comparison was made between those affected and the remainder of the local population.

The purpose of this present study therefore was to assess the relative impact of filarial lymphoedema on quality of life using a case-control paradigm. The study primarily focuses on the physical impact of the condition, and uses both subjective and objective measures to compare participants’ perceived and observed mobility in both test and natural settings. A secondary aim was to obtain information on income and health expenditure in order to make comparison between the two groups.

This study provides valuable information to national LF programmes and their donors in an approach that can be used in quantifying the impact of LF morbidity. Further, it provides a framework that can be used to assess the benefit of future morbidity interventions e.g. lymphoedema management activities or clinical drug trials [17].

## Methods

Individuals affected by LF clinical conditions from two health centre catchment areas (Nchalo and Bereu) in Chikwawa district, Southern Region, Malawi were included in this study, which was conducted in May 2015. Clinical cases were identified and recruited based on data from a previous morbidity mapping study in March 2014 [14], which identified 54 lymphoedema patients (36 in Nchalo, 18 in Bereu). A total of 31 lymphoedema patients included in this study to ensure that the study had sufficient power (80%) to detect a mean quality of life (QoL) score difference (described below) as little as 8 between cases and controls using a significance level of 5%. Stratified random sampling was used to select the 31 cases, using catchment area and severity of condition as strata i.e. strata of Dreyer stages 1–4 (44/54 previously identified patients) and 5–7 (10/54 previously identified patients) as assessed during case identification. All forms of identified lymphoedema (leg, arm, and breast) were considered for this part of the study. One control per case was then selected, matched by village, sex and approximate age. Population registers maintained by the senior health surveillance assistant (HSA) of each catchment area were used to select a minimum of four potential controls per case of which one was randomly selected for inclusion in the study.

### Quality of Life (QoL) questionnaire

Each case and matched control completed a self-assessed QoL questionnaire to determine their physical and psychological health, their ability to care for themselves and undertake regular activities, and their economic well-being (see Additional file 2). The questionnaire was adapted from the LF-specific Quality of Life Questionnaire (LFSQQ) derived by Aggithaya et al. (2013) to assess lymphoedema patients in India [18]. Modifications were made to this questionnaire in consultation with local health workers in order to ensure that the questions were culturally relevant. For example, the original questionnaire included separate questions as to whether they experienced any difficulty in using an Indian toilet or a European toilet. This was altered to a single question to determine whether participants had any difficulty using a local toilet. Participants were asked to assess their ability to complete a list of tasks on a scale of 0 (no problem completing the task) to 4 (extreme problem completing the task). There were five tasks for each of the five QoL categories (mobility, self-care, usual activities, psychological health, social participation), resulting in a total of 25 questions. The overall QoL score was calculated as a percentage by summing all the scores and dividing this total by 4 × Number of questions answered. Comparisons of the overall QoL scores, and scores obtained in each of the five categories (sum of individual category scores/ [4 × Number of questions answered]), were made between cases and controls to assess the perceived relative impact of lymphoedema. Each case was also asked questions about their condition including the number of acute attacks they had experienced in the previous 6 months, how long the attacks usually lasted, and whether they felt their lymphoedema affected their ability to work.

### Timed mobility tests

In addition to measuring perceived mobility problems on the 0 to 4 scale (sitting or getting out of a chair, lying down or standing up from the floor, going up steps, walking for one hour without a break, using public transport), objective measures of mobility were recorded. Each participant completed a 10 m walking test (10MWT) [19] and a timed ‘up and go’ (TUG) test [20, 21] which measure gait speed and balanced walking ability respectively. The 10MWT required participants to walk for 10 m from a static start without assistance, and the time taken to walk the intermediate 6 m was recorded. Assistive devices were allowed if required, and participants were instructed to walk at a comfortable and safe speed. The timed test was repeated three times sequentially with a short break (~1 min) between each test, and the average time was recorded. The TUG test, which was undertaken immediately after the 10MWT, required participants to start from a seated position, and the time it took for the participant to stand up, walk to a point three metres from the chair, turn around, walk back to the chair then sit down was recorded. As with the 10MWT, participants were asked to walk at a comfortable speed, the test was repeated three times sequentially with a short break in between, and the average time was recorded. Average times were compared between cases and controls to obtain a more objective assessment of relative impact of lymphoedema on mobility. The Pearson correlation coefficient between the self-assessed scores and the timed tests were calculated to determine differences in perceived and actual disability.

### Natural mobility measures

A subset of 10 matched pairs, with a focus on leg lymphoedema only, also had their mobility measured in a more natural environment over a period of 3 weeks using GPS data loggers, specifically the i-gotU GT-120 (http://www.i-gotu.com/). Each participant was instructed to wear the device on their arm during waking hours, and the device recorded their GPS location every 60 s. Participants were visited by the research team every other day throughout a 3 week period (excluding Sunday) during which the GPS data logger was swapped for a fully charged device, and the previous 2 days of data was downloaded and stored. The data were cleaned by removing any single points that were clearly inaccurate, and further excluding points during which it appeared that public transport (bicycle, minibus) was being used i.e. if their speed exceeded 6.5 km/h. Further, each point was categorised as either stationary or walking based on a speed threshold of 0.5 km/h. Minimum convex polygons and kernel density estimation were used to identify each participant’s ‘activity area’ or ‘home range’ [22]. The minimum convex polygon is the smallest possible convex polygon that encloses all (or a subset of) the recorded GPS points. This method is commonly used to assess the home range of animals, and assumes that the subject of interest moves around the entire area indiscriminately. However, as people tend to move along defined routes e.g. roads, pathways, this method may not be a true representation of the area around which human subjects move. Kernel density smoothing results in a smoothed surface representing the density of recorded GPS points over the area of interest [22, 23]. Using this method, the 99% activity area is estimated as the smallest area over which the kernel density sums to 0.99. The minimum convex polygon home range and the kernel density activity area were calculated using the R package adehabitatHR [24], and maps of the results were created in QGIS version 2.12.0 [25], using OpenStreetMap data (openstreetmap.org) as a base map.

Summaries of mobility were then calculated and comparisons made between cases and control using the non-parametric Wilcoxon signed-rank test i.e. the average daily moving distance, average daily walking distance, average walking speed and size of activity area. To determine whether there was any consistency between the self-assessed mobility scores, the timed mobility tests, and the GPS results, the Kendall rank correlation coefficient, commonly known as Kendall’s tau, was calculated for both cases and controls [26, 27]. Kendall’s tau is a non-parametric test for correlation that is similar to Spearman’s rank, but is less sensitive to outliers. Kendall’s tau ranges between +1 (perfectly matched ranking) and −1 (fully discordant ranking).

### Income and health expenditure

In addition to completing the self-assessed QoL questionnaire, all 31 cases and controls were also asked to provide socioeconomic information that included their employment, average weekly earnings and average weekly health expenditure. Paired t-tests were performed to compare earnings and health expenditure between the two groups. As questionnaire responses were subject to recall bias, all 10 cases and controls participating in the GPS data logger exercise were also asked to record their daily earnings and health expenditure over the 3 week period. Due to the small sample size, the Wilcoxon signed-rank test was again used to compare the two groups.

## Results

Of the 31 cases and control pairs participating in the main study (20 in Nchalo, 11 in Bereu), 8 (26%) pairs were male and 23 (74%) pairs were female with cases and controls having a mean age of 58 and 55 respectively. Of the 31 cases, 22 had lymphoedema in one leg, 4 had lymphoedema in both legs, 3 had lymphoedema in one arm and 2 had lymphoedema in one arm and one leg. Employment levels were similar between the two groups (77.4% [24/31] cases, 74.2% [23/31] control), with all 47 employed participants reporting to be farmers.

### QoL questionnaire

With regards to the self-assessed QoL questionnaire, cases had a mean QoL score of 32.2 (sd = 14.1) in comparison to 6.0 (sd = 10.9) for controls, with the average difference of 26.2 being statistically significantly different (*P* < 0.01, 95% *CI* = [20.9, 31.4]). Table 1 presents these results, in addition to the individual category scores for cases and controls. Significant differences in scores were seen in all five categories, with the largest difference occurring in mobility (43.1 in cases, 7.4 in controls, *P* < 0.01) and psychological health (40.8 in cases, 9.8 in controls, *P* < 0.01), followed closely by usual activities (35.9 in cases, 5.8 in controls, *P* < 0.01). There was generally more variability in the responses of cases in comparison to controls, particularly with regards to the average mobility score (sd = 25.3 for case, 2.7 for controls). In comparing the scores of the severe cases (6/31) to that of the less severe cases (25/31), only a small difference was observed between the two groups (severe: mean mobility score = 48.3, sd = 23.4; less severe: mean mobility score = 41.8, sd = 26.1) which may explain some of this variability, however the difference in within-group variability was still considered to be large. With regards to the additional questions directed to cases only, all but two (94% of 31 responses) had experienced at least one acute attack in the past 6 months, with the number of attacks ranging from one to ‘uncountable’. Of the 26 that reported an exact number, the median value was 2. When asked whether they felt that their lymphoedema affected the number of hours they were able to work, 92% of respondents (24/26) believed that it did. Of the remaining two, one had a moderate lymphoedema (both legs affected) and one had a severe lymphoedema (one leg affected).

**Table 1:** Summary of scores obtained using the self-assessed QoL questionnaire for cases and control

Category	Cases		Control		95% *CI* of difference	*P*-value
	Mean	SD	Mean	SD		
Overall	32.2	14.1	6.0	10.9	20.9–31.4	<0.01
Mobility	43.1	25.3	7.4	2.7	26.1–45.2	<0.01
Self-care	16.3	15.0	1.0	9.8	9.7–20.9	<0.01
Usual activities	35.9	19.8	5.8	6.6	22.9–37.4	<0.01
Psychological health	40.8	17.2	9.8	5.6	24.3–37.7	<0.01
Social participation	25.0	12.1	6.3	5.9	13.8–23.6	<0.01

### Timed mobility tests

The results of the 10MWT and the TUG tests (Table 2) showed on average, cases are slower than controls in both of these tests (10WT: cases = 0.828 m/s (sd = 0.216), controls = 1.104 m/s (sd = 0.303), *P* < 0.01; TUG: cases = 14.7 s (sd = 5.500), controls = 11.2 s (sd = 3.622), *P* < 0.01). It was also noted that males were marginally quicker than females (see Additional file +2). In comparing the cases, more severe cases were on average slower than less severe cases in both the 10MWT (severe: mean = 0.734 m/s, sd = 0.258; less severe: mean = 0.850 m/s, sd = 0.204) and the TUG tests (severe: mean = 16.1, sd = 7.189; less severe: mean = 14.3 s, sd = 5.141), although neither of the differences were significant, possibly due to the small sample size.

**Table 2:** Summaries of the timed mobility tests (10 m walking test speed, and time to complete the timed up and go test)

Timed tests		Mean	SD	95% *CI* of difference	*P*-value
10 m walking test (m/s)	Cases	0.828	0.216	0.165–0.387	<0.01
	Controls	1.104	0.303		
Timed up and go (secs)	Cases	14.7	5.500	−5.354, −1.731	<0.01
	Controls	11.2	3.622		

**Fig. 1:**
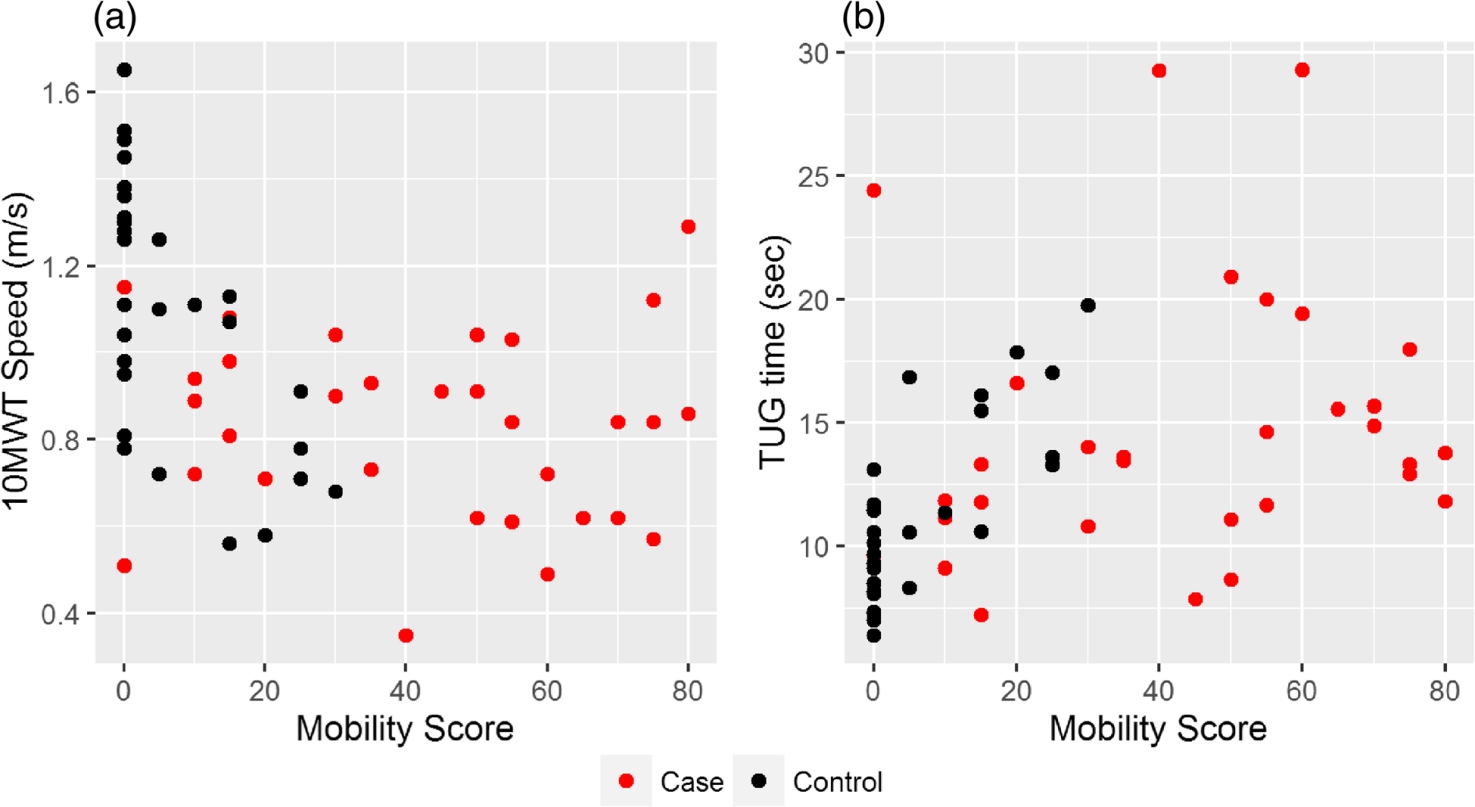
Scatterplots of the results of the mobility score against the *10MWT speeds* **a** and *TUG* test times **b**. Cases and controls are represented by *red* and *black dots* respectively

Table 3 presents the correlation between perceived QoL (both overall score, and mobility score) and the results of the 10MWT and the TUG tests. The relationship between the mobility tests and the perceived QoL in controls was consistent for both tests when considering both the overall QoL score and the mobility score only. With regards to the cases, there was a very weak, non-significant negative relationship observed between the 10MWT and the overall scores (correlation = −0.12, 95% *CI* = (−0.45, 0.25)) and mobility scores (correlation = −0.06, 95% *CI* = (−0.41, 0.30)), whereas a slightly stronger, yet still non-significant negative correlation was observed between the TUG test and the overall QoL scores (correlation = 0.30, 95% *CI* = (−0.06, 0.59)) and mobility scores (correlation = 0.18, 95% *CI* = (−0.19, 0.50)) (Fig. 1). This indicates that self-assessed physical disability differed from objectively measured physical disability in the cases, whereas the two measures were consistent for the controls.

**Table 3:** Correlation between the self-assessed disability and mobility scores and the timed mobility tests

Test		Overall disability score		Mobility score	
		Correlation	95% *CI*	Correlation	95% *CI*
10 m walking test (m/s)	Cases	−0.12	−0.45, 0.25	−0.06	−0.41, 0.30
	Controls	−0.63	−0.80, −0.36	−0.61	−0.79, −0.34
Timed up and go (secs)	Cases	0.30	−0.06, 0.59	0.18	−0.19, 0.50
	Controls	0.75	0.52, 0.87	0.77	0.57, 0.88

### Natural mobility measures

**Fig. 2:**
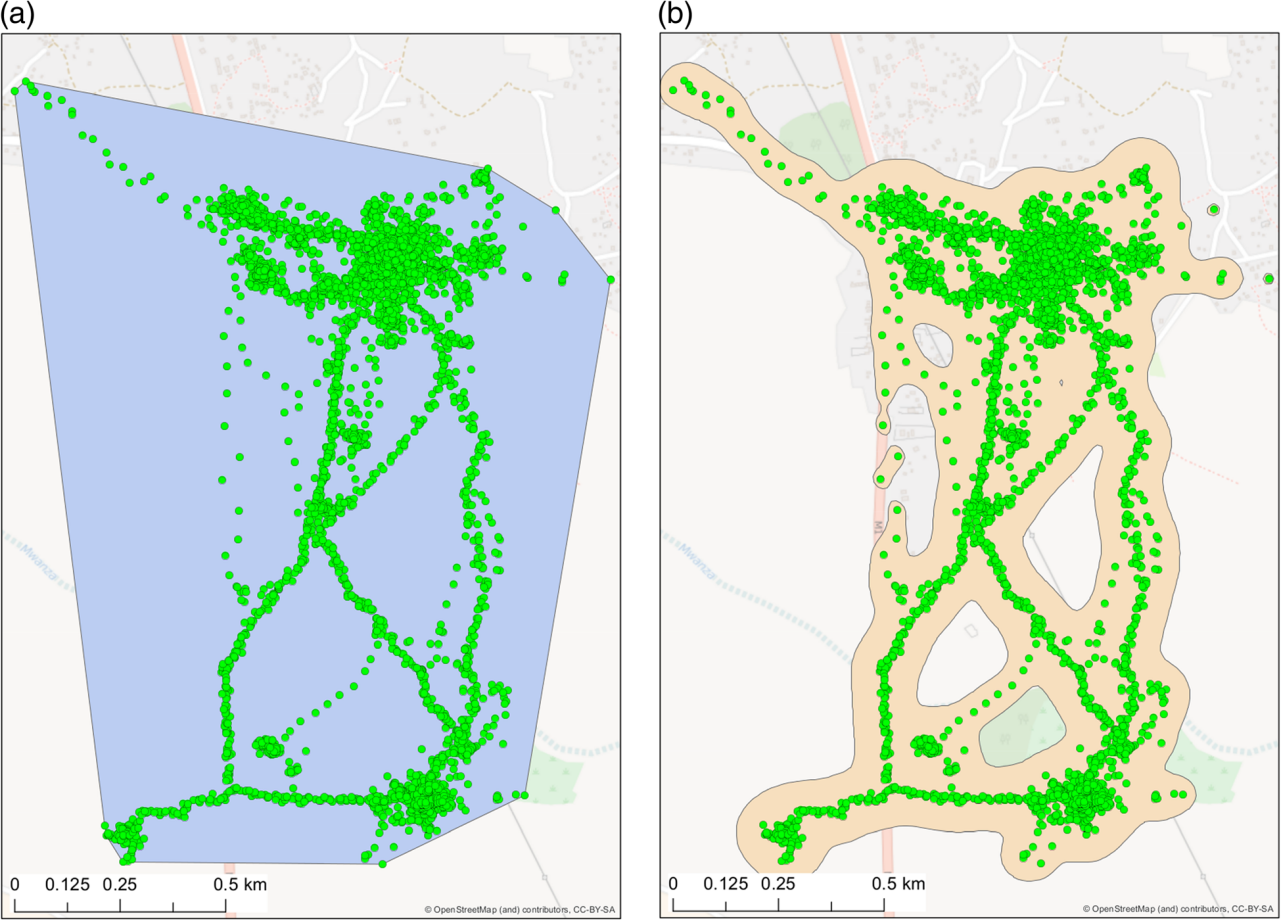
Example GPS points for one participant: **a** activity area for the participant as a minimum *convex polygon*, **b** activity area obtained using 99% *kernel density* smoothing of the GPS points

Summaries of the GPS data, recorded for 10 cases and 10 controls over approximately a 3 week period (20 days on average for cases, 21 days for controls), are presented in Table 4. The minimum convex polygon activity area was calculated using all GPS coordinates collected over this time period. The kernel density activity area was calculated using a 10 m resolution grid, and the value of the smoothing parameter h was obtained using the median “reference” bandwidth calculated using the cases data only i.e. h = 30. Figure 2 presents a visualisation of these results for one participant. In this example 28.279 points were collected over a 24 day period, and the resulting minimum convex polygon activity area was 1.85 km^2^, whereas the 99% kernel density activity area was 1.05 km^2^. No significant differences between cases and controls were observed between any of the summary measures under consideration, however the median value for the cases was consistently lower than that of the controls i.e. cases moved shorter distances (median = 16.28 km/day for cases vs 19.23 km/day for controls) and moved at a slower speed (median = 0.186 m/s for cases vs 0.209 m/s for controls). The activity area for cases was generally smaller than that of controls using both measurement methods i.e. the median for cases was 4.30 km^2^ in comparison to 7.14 km^2^ for controls using the minimum convex polygon method, and 1.25 km^2^ for cases in comparison to 2.10 km^2^ for controls using the kernel smoothing method.

**Table 4:** Summaries of the GPS data logger measurements for cases and controls

Timed tests		Median	Q1	Q3	*P*-value
Average distance (km/day)	Cases	16.28	14.97	19.41	0.375
	Controls	19.23	16.38	20.16	
Average speed (m/s)	Cases	0.186	0.172	0.237	0.625
	Controls	0.209	0.183	0.244	
Home range (minimum convex polygon) km^2^	Cases	4.30	0.84	23.16	0.375
	Controls	7.14	4.60	19.92	
99% Home range (kernel smoothing) km^2^	Cases	1.25	0.51	2.75	0.160
	Controls	2.10	1.11	3.09

Table 5 presents the value of Kendall’s tau between the two activity area measures and the self-assessed QoL scores and timed mobility tests. No significant associations were observed between the self-assessed overall QoL and mobility scores and the home range measures, although may be as a consequence of the small sample size. With regards to the timed mobility tests, (borderline) significant correlation was observed between both activity area measures for the 10MWT (MCP: tau = 0.45, 95% *CI* = (0.030, 0.869); KS: tau = 0.58, 95% *CI* = (0.193, 0.976)) and the TUG test (MCP: tau = −0.42, 95% *CI* = (−0.792, −0.048); KS: tau = −0.47, 95% *CI* = (−0.933, −0.001)), whereas there were no significant relationships observed for the controls. This is in contrast to the relationships observed between the self-assessed QoL scores and the timed mobility tests, in which the results for the controls were significant, but with the cases they were not.

**Table 5:** Kendall’s rank correlation (Kendall’s tau) between activity area measures and self-assessed score and timed mobility test results

		Minimum convex polygon activity area		Kernel smoothing activity area	
		Correlation (tau)	95% *CI*	Correlation (tau)	95% *CI*
Disability score	Cases	−0.02	−0.592, 0.547	−0.15	−0.678, 0.360
	Controls	−0.05	−0.686, 0.594	−0.05	−0.530, 0.438
Mobility score	Cases	−0.04	−0.481, 0.391	−0.13	−0.666, 0.396
	Controls	0.11	−0.355, 0.566	−0.11	−0.566, 0.355
10 m walking test (m/s)	Cases	0.45	0.030, 0.869	0.58	0.193, 0.976
	Controls	0.31	−0.163, 0.792	0.31	−0.235, 0.864
Timed up and go (m/s)	Cases	−0.42	−0.797, −0.048	−0.47	−0.933, −0.001
	Controls	−0.38	−0.767, 0.011	−0.38	−0.900, 0.145

### Income and health expenditure

In comparing the questionnaire responses of the 31 cases and control, overall the two groups were similar in employment (77% [24/31] of cases, 74% [23/31] of controls), however, on average, cases were unable to work for 17 days over the previous 3 months on average (min = 0, max = 60), whereas no controls reported being unable to work (*P* < 0.01). The average self-reported weekly earnings were lower in cases (708MK vs 1 259MK, *P* = 0.364), although this difference was not statistically significant. Further, there was a non-significant difference in the average amount spent on health in the previous 3 months (cases = 3 383MK, controls = 2 258MK, *P* = 0.363), with 6 cases (20%, 30 responses) and 8 control (26%, 31 responses) reporting that they accessed free government healthcare only.

With regards to the daily income and expenditure data collected during the GPS data logger exercise (Table 6), median weekly earnings for cases was close to three times greater than that of controls (cases = 527MK, controls = 1 393MK, *P* = 0.064), with this difference being borderline significant. This differed from the self-reported weekly earnings provided by this subset of participants (cases = 500MK, controls = 800MK) during the questionnaire. In comparing median weekly health expenditure, cases spent less than controls in both absolute (cases = 147MK, controls = 278MK, *P* = 0.359) and relative terms (cases % of earnings = 16%, controls % of earnings = 22%, *P* = 0.461) although neither of these differences were significant.

**Table 6:** Reported weekly earnings and health expenditure of 10 cases and controls over a 3 week period

	Cases		Controls	
	Median	Range	Median	Range
Median weekly earnings (MK)	527	261–1 046	1 393	451–3 582
Median weekly health expenditure (MK)	147	0–404	278	9–1 578
% weekly earnings spent on health	16	0–41	22	2–68

## Discussion

This study contributes to the growing body of evidence on the physical, social and economic impact of lymphoedema [2, 15], and in particular is one of the few studies that objectively quantifies the relative impact of lymphoedema on mobility, in comparison to an unaffected cohort of controls with a similar age distribution and occupational status. It is clear from this study that those affected by lymphoedema are at a disadvantage to their unaffected peers, experiencing a lower quality of life, particularly with regards to their level of mobility, as confirmed by both the subjective QoL survey responses and objective timed test results and GPS data collected during this study. To the authors’ knowledge, this is the first time that the latter two approaches have been used to measure mobility in lymphoedema cases in LF-endemic areas. This study further supports previous work of the economic consequences of lymphoedema, or neglected tropical diseases (NTDs) in general [6, 7, 28], with controls’ median weekly earnings over the 3 week study period (1 393MK, ~$1.86) being three times greater than that of cases (527MK, ~$0.70).

With regards to the measures used, this study indicates that objective measures of physical disability such as the 10 m walking test and the timed up and go test are a useful supplement to QoL questionnaires when assessing the impact of LF morbidity management interventions. These two measures were uncorrelated in cases, indicating that self-assessed disability and measured disability differed in this cohort. It may therefore be the case that another aspect of the lymphoedema is having a greater impact on the case’s perceived disability, for example the frequency or duration of acute attacks. This lack of a relationship between the QoL score (both overall and mobility) for the cases therefore indicates that when assessing the physical impact of lymphoedema, multiple measurements should be considered to obtain an accurate picture of the impact in terms of both perceived and measurable disability. Further, detailed longitudinal mobility data such as that obtained by the GPS data logger may allow assessments of physical ability to be made in a more natural setting. This study indicates that the activity area measure is most closely associated with mobility under test conditions for cases only, whereas no association was observed for controls; thus further work is needed to determine how GPS data can best be used to assess mobility improvements. Whilst the use of the GPS data loggers was labour intensive due to the need to frequently charge the devices, advances in GPS-enabled wearable technologies will likely make this level of assessment more accessible in the future [29].

## Conclusions

This study highlights the importance of ensuring that continued efforts be made to address the negative health, social and economic impacts of filarial lymphoedema and their associated acute attacks. Appropriate quality of life and mobility measures such as those described in this study will enable the effectiveness of future clinical and other therapeutic interventions to be evaluated, and the full benefit of morbidity management activities to be realised.

## Additional files

Additional file 1: Multilingual abstract in the five official working languages of the United Nations. (PDF 835 kb)

Additional file 2: Case-control Questionnaire. (DOCX 21 kb)

## Abbreviations

10MWT: 10 m walking test
ADLA: Acute dermatolymphangioadenitis
GPELF: Global Programme to Eliminate Lymphatic Filariasis
HSA: Health Surveillance Assistant
LF: Lymphatic filariasis
QoL: Quality of life
TUG: Timed up and go
