# Supplementary material for: Measuring the physical and economic impact of filarial lymphoedema in Chikwawa district, Malawi: a case-control study
Source: Infect Dis Poverty. 2017 Apr 3;6:28. doi: 10.1186/s40249-017-0241-2 (PMC5376674; doi:10.1186/s40249-017-0241-2)
Supplement: Supplementary file 1 — Multilingual abstract in the five official working languages of the United Nations. (PDF 835 kb) [file 40249_2017_241_MOESM1_ESM.pdf]

Translation of the abstract into the five official working languages of the United Nations

قياس الأثر المادي والاقتصادي للوذمة اللمفية الفيلارية في منطقة شيكواوا، ملاوي: دراسات حالة مقارنة

ميشيل جيم ستانتون، ماساتو ياموتشي، ساحة ز. مكواندا، بول ندلوفو، دوروثي إيمي ماتيبولا، تشارلز ماكنزي، لويز كيلي هوب

ملخص

خلفية: داء الخيطيات اللمفاوية (LF) هو أحد الأسباب الرئيسية للوذمة اللمفية في أفريقيا جنوب الصحراء الكبرى، وله تأثير كبير على نوعية الحياة للمتضررين. في هذا البحث تقييم للأثر النسبي للوذمة اللمفية على التنقل والدخل في منطقة شيكواوا في ملاوي.

الطرق: أكملت عينة عشوائية من 31 شخصا مصابين بالوذمة و31 يمثلون مجموعة ضابطة ملائمة استبيانا حول نوعية الحياة الذي حسبت منه النتيجة بشكل عام ووفقا لدرجة الحركة على حد سواء. أجريت تجربتين للحركة، وهما اختبار المشي لعشرة أمتار واختبار سرعة القيام من الكرسي، وارتدت مجموعة فرعية ضابطة من 10 حالات مسجلات بيانات مربوطة بنظام التموضع العالمي لمدة ثلاثة أسابيع لقياس قدرتهم على الحركة في وضع أكثر طبيعية. تم جمع البيانات الاقتصادية بأثر رجعي من جميع الحالات الـ31 في المجموعة التجريبية والحالات المناظرة في المجموعة الضابطة، وكل مشارك ارتدى مسجلات البيانات المربوطة بنظام التموضع العالمي سجَّل نشاطه اليومي ونفقاته الصحية خلال فترة المراقبة.

النتائج: كانت نوعية الحياة للمجموعة التجريبية أفقر بصفة عامة وبشكل ملحوظ (المجموعة التجريبية = 32.2، المجموعة الضابطة = 6.0، P<0.01) والنتائج المرتبطة بالحركة (المجموعة التجريبية = 43.1، المجموعة الضابطة = 7.4، P<0.01) بالمقارنة مع المجموعة الضابطة. وكانت المجموعة التجريبية أيضا أبطأ بكثير (P <0.01) في استكمال اختبارات الحركة في توقيتها، على سبيل المثال، سرعة السير في اختبار السير لمدة عشر دقائق 0.83 م/ث للمجموعة التجريبية بالمقارنة مع 1.10 م/ث للمجموعة الضابطة. وقد لوحظ وجود علاقة متناسقة بين نتائج نوعية الحياة-الحركة المحددة وتوقيت نتائج اختبار المجموعة التجريبية (السير لمدة عشر دقائق الارتباط = -0.06، 95٪ CI= (-0.41، 0.30))، مشيرا إلى أن إعاقتهم المنظورة تختلف عن إعاقتهم المقاسة، في حين وكانت النتائج متسقة مع المجموعة الضابطة (السير لمدة عشر دقائق الارتباط = -0.61، 95٪ CI = (-0.79، -0.34)). وأشارت سجلات تحديد المواقع أن المجموعة التجريبية تسير عموما مسافات أقصر بسرعة أبطأ من المجموعة الضابطة، وتغطي مساحة جغرافية صغيرة (منطقة متوسط بأساليب التمهيد: المجموعة التجريبية = 1.25 كيلو متر مربع، المجموعة الضابطة = 2.10 كيلو متر مربع، P= 0.16). ذكرت المجموعة التجريبية أنهم يكسبون أقل من نصف ما يكسبه المجموعة الضابطة في الأسبوع (المجموعة التجريبية = 0.70 دولار، المجموعة الضابطة = 1.86 دولار P = 0.064)، وينفقون نسبة أقل من دخلهم (16٪ مقابل 22٪، P= 0.461) على الرعاية الصحية.

الاستنتاجات: كان المصابون بالوذمة في وضع غير مؤات مقارنة بأقرانهم الأصحاء، حيث يعانون من تدني نوعية الحياة كما أكدت كل من اختبارات الحركة الذاتية والموضوعية، ومن انخفاض الدخل. وتشير هذه الدراسة أيضا إلى أن الاختبارات الموضوعية للحركة قد تكون تكملة مفيدة لاستبيانات التقييم الذاتي لنوعية الحياة عند تقييم التأثير المستقبلي لتدخلات السيطرة على الوذمة.

Translated from English version into Arabic by Mahmoud Sami, through

TRANSLATORS
WITHOUT BORDERS

评价马拉维奇克瓦瓦县丝虫性淋巴水肿对患者机体和经济影响的一项病例对照研究

Michelle C. Stanton, Masato Yamauchi, Square Z. Mkwanda, Paul Ndhlovu, Dorothy Emmie Matipula, Charles Mackenzie and Louise Kelly-Hope

摘要
引言：淋巴丝虫病(LF)是造成撒哈拉以南非洲地区淋巴水肿的主要原因之一，同时它对淋巴丝虫病患者的生活质量影响巨大。本研究评估了马拉维奇克瓦瓦县淋巴水肿对患者行动和收入的影响。
方法：随机抽样 31 例淋巴水肿患者和 31 例匹配的对照患者完成生活质量问卷调查，并计算总体评分和行动特异性评分。通过两项迁移测试，10 m 行走测试[10MWT]和起立-行走时间测试[TUG]，以及包括对 10 对病例-对照患者进行连续三周穿戴 GPS 数据记录器的测试，在更接近自然环境中测定他们的行动能力。收集 31 对病例-对照病例的回顾性经济数据，并且记录整个观察期间每个参与 GPS 活动监测的受试者的收入和卫生支出情况。
结果：与对照组相比，病例组总体生命质量（病例组: 32.2，对照组: 6.0，*P*<0.01）和行动特异性的分数（病例组: 43.1，对照组: 7.4，*P*<0.01）明显较低。在完成定时迁移测试时，病例组也明显较慢（*P*<0.01），病例组平均 10 m 行走速度为 0.83 m/s，而对照组为 1.10 m/s。在病例组的行动特异性评分和定时迁移测试结果不一致 [10 MWT correlation=-0.06，95% *CI*=（-0.41, 0.30）]，这表明他们的感知到的障碍与测量的残疾不同，而此项结果在对照组中的关系也是一致的[10 MWT correlation=-0.61, 95% *CI*=（-0.79, -0.34）]。GPS 结果表明，一般情况下，病例组走较短的距离比对照组速度慢，并且仅覆盖一个较小的地理区域（内核平滑中值区域：病例组为 1.25 km^2，对照组为 2.10 km^2，*P*=0.16）。病例组每周收入不到对照组的一半（病例组= 0.70 美元，对照组= $ 1.86，*P*=0.064），并且在医疗保健上的支出也较小（病例组=16%，对照组=22%，*P*=0.461）。
结论：淋巴水肿患者与未感染者相比具有明显的劣势，其生活质量较低，并且主观和客观的行动性评价和较低的收入均证实了这一点。本研究还表明，在评估淋巴水肿管理干预措施的未来影响时，关于行动性的客观评价可能是对自我评价生命质量问卷的一个有效补充。

Translated from English version into Chinese by Xin-Yu Feng, edited by Pin Yang， through

CHINESE CENTER FOR DISEASE CONTROL AND PREVENTION
NATIONAL INSTITUTE OF PARASITIC DISEASES

Mesure de l'impact physique et économique du lymphœdème dû à la filariose dans le district de Chikwawa au Malawi : étude cas-témoin

Michelle C. Stanton, Masato Yamauchi, Square Z. Mkwanda, Paul Ndhlovu, Dorothy Emmie Matipula, Charles Mackenzie et Louise Kelly-Hope

Résumé
Contexte : La filariose lymphatique est l'une des principales causes de lymphœdème en Afrique subsaharienne. Son impact sur la qualité de vie des personnes affectées est considérable. Nous évaluons ici l'impact relatif du lymphœdème sur la mobilité et les revenus dans le district de Chikwawa, au Malawi.
Méthodes : Un échantillon randomisé de 31 sujets atteints de lymphœdème et 31 témoins assortis a rempli un questionnaire qui a été utilisé ensuite pour calculer un score général de qualité de vie (QDV) et un score spécifique de mobilité. Deux tests de mobilité ont été réalisés : une épreuve de marche sur 10 mètres (10MWT) et une épreuve de départ assis chronométré (TUG). Un sous-ensemble de 10 paires de cas et de témoins a été doté d'enregistreurs de données GPS pendant trois

semaines, afin de mesurer leur mobilité dans un cadre plus naturel. Des données économiques rétrospectives ont été recueillies auprès des 31 paires de cas et témoins et chaque participant équipé d'un GPS a enregistré ses gains journaliers et ses dépenses de santé pendant toute la période d'observation.

Résultats : Les scores étaient significativement moins bons pour les cas que pour les témoins, qu'il s'agisse du score général de qualité de vie (cas = 32,2, témoins = 6,0, $P < 0{,}01$) ou du score spécifique de mobilité (cas = 43,1, témoins = 7,4, $P < 0{,}01$). Les cas se sont aussi avérés significativement plus lents ($P < 0{,}01$) dans les épreuves de mobilité chronométrées, avec par exemple une vitesse moyenne de marche sur 10 mètres de 0,83 m/s contre 1,10 m/s pour les témoins. Une relation inconstante a été observée entre les scores de QDV concernant spécifiquement la mobilité et les résultats des épreuves chronométrées pour les cas (corrélation pour 10MWT = -0,06, IC à 95 % = (-0,41, 0,30)), ce qui indique que leur handicap perçu était différent du handicap mesuré, tandis que les résultats étaient constants pour les témoins (corrélation pour 10MWT = -0,61, IC à 95 % = (-0,79, -0,34)). Les relevés de GPS indiquent que les cas marchent généralement sur de plus courtes distances et moins vite que les témoins et couvrent une moins grande zone géographique (aire médiane par lissage de noyaux : cas = 1,25 km^2, témoins = 2,10 km^2, $P = 0{,}16$). Les cas ont rapporté un revenu par semaine inférieur à celui des témoins (cas = 0,70 $, témoins= 1,86 $, $P = 0{,}064$) et consacraient une plus petite part de ces recettes à leurs soins de santé (16 % contre 22 %, $P = 0{,}461$).

Conclusions : Les patients atteints de lymphœdème sont clairement désavantagés par rapports à des sujets comparables non atteints. Leur qualité de vie est moins bonne, comme le montrent des mesures subjectives et objectives de la mobilité, et leurs revenus sont inférieurs. Cette étude indique également que les mesures objectives de la mobilité pourraient être un complément utile aux questionnaires auto-administrés sur la qualité de vie pour évaluer l'impact futur des interventions de lutte contre le lymphœdème.

Translated from English version into French by Suzanne Assenat, through

TRANSLATORS
WITHOUT BORDERS

Оценка физического и экономического воздействия филярийной лимфедемы в округе Чиквава, Малави: исследование методом случай-контроль

Мишель С. Стэнтон (Michelle C. Stanton), Масато Ямаючи (Masato Yamauchi), Сквэа З. Мкванда (Square Z. Mkwanda), Пол Ндхлову (Paul Ndhlovu), Дороти Эмми Матипула (Dorothy Emmie Matipula), Чарльз Маккензи (Charles Mackenzie) и Луиз Келли-Хоуп (Louise Kelly-Hope)

Аннотация

Краткое описание. Лимфатический филяриатоз (ЛФ) является одной из главных причин лимфатических отёков в Чёрной Африке и сильно сказывается на качестве жизни больных. В

этой статье мы оцениваем относительное влияние лимфедемы на подвижность и уровень доходов в округе Чиквава, Малави.

Методы. Выбранные случайным образом 31 больной лимфедемой и 31 человек из аналогичной контрольной группы заполнили опросник оценки качества жизни, по которому были рассчитаны оценка общего качества жизни и оценка подвижности. Были проведены два теста на подвижность, а именно: ходьба на 10 м [10МХ] и тест на время [ТНВ]. Кроме того, подгруппа из 10 пар случай-контроль носила регистраторы данных GPS в течение трёх недель для измерения их подвижности в более естественной среде. Были собраны ретроспективные экономические данные всех 31 пары случай-контроль, и все участники измерения активности с помощью GPS записывали свой ежедневный заработок и расходы на медицинское обслуживание на протяжении периода наблюдения.

Результаты. У больных по сравнению с контрольной группой были значительно ниже общая оценка качества жизни (случаи = 32,2, контрольная группа = 6,0, P < 0,01) и оценка подвижности (случаи = 43,1, контрольная группа = 7,4, P < 0,01). Больные были также заметно медленнее (P < 0,01) при выполнении тестов подвижности на время, напр., их средняя скорость теста 10МХ составила 0,83 м/с в сравнении с 1,10 м/с у контрольной группы. Была отмечена несогласованность между оценками качества жизни в отношении подвижности и результатами тестов на время у больных (корреляция 10МХ = -0,06, 95%*CI* = (-0,41, 0,30)), что указывает на то, что их предполагаемая инвалидность отличается от измеренной; при этом результаты контрольной группы соответствовали ожиданиям (корреляция 10МХ = -0,61, 95%*CI*= (-0,79, -0,34)). Сводные данные GPS показали, что больные, как правило, проходят более короткие расстояния и делают это медленнее, чем контрольная группа, покрывая при этом меньшую географическую территорию (средняя площадь по методу ядерного сглаживания: случаи = 1,25 км^2, контрольная группа = 2,10 км^2, P = 0,16). Заработок больных в неделю составил меньше половины заработка контрольной группы (случаи = $0,70, контрольная группа = $1,86, P = 0,064). Они истратили на медицинское обслуживание меньшую часть своего заработка (16% в сравнении с 22%, P = 0,461).

Заключение. Больные лимфедемой явно находятся в менее выгодном положении по отношению к незаражённым лицам. У них более низкая оценка качества жизни, что подтверждено субъективными и объективными измерениями подвижности, и более низкий доход. Данное исследование также показало, что объективные измерения подвижности могут быть полезной дополнительной информацией к самостоятельно заполняемым опросникам при оценке эффективности мер по борьбе с лимфедемой в будущем.

Translated from English version into Russian by Natalia Potashnik, through

TRANSLATORS
WITHOUT BORDERS

Evaluación del impacto físico y económico de la filariasis linfática en el distrito de Chikwawa en Malawi: Un estudio de casos y controles

Michelle C. Stanton, Masato Yamauchi, Square Z. Mkwanda, Paul Ndhlovu, Dorothy Emmie Matipula, Charles Mackenzie and Louise Kelly-Hope

Resumen

Historial: La Filariasis Linfática (FL) es una de las causas primarias de linfedema
en la region de África subsahariana, y tiene un impacto significativo en la calidad de vida de aquellas personas afectadas. En este artículo evaluamos el impacto de linfedema tanto en la movilidad física como en los ingresos económicos de los pacientes en el distrito de Chikwawa, Malawi.

Métodos: Una muestra aleatoria de 31 personas con linfedema y 31 controles comparados; completaron un cuestionario sobre la calidad de sus vidas, ambos se calcularon sobre una puntuación general de movilidad específica.

Se realizaron dos pruebas de movilidad, específicamente las pruebas de caminata de 10 m [10PDC] y la cronometrada de levántate y anda (Timed Up and Go – TUG test) y, un subgrupo de 10 pares de casos controles llevaron consigo registradores de datos GPS durante tres semanas con el fin de medir su movilidad en un entorno más natural. Además se recogieron todos los datos retrospectivos económicos de las 31 pares de casos controles, y también los datos de cada uno de los participantes que registraron su actividad diaria GPS y sus gastos de salud durante el período de observación.

Resultados: En conjunto, los casos presentaron una calidad de vida significatimente más pobre (casos = 32.2, controles = 6.0, $P < 0.01$) y los de movilidad específica (casos = 43.1, controles = 7.4, $P < 0.01$) puntuaciones en comparasión a los de controles. Además, los casos fueron significativamente más lentos ($P < 0.01$) al completar las pruebas cronometradas de movilidad, ej.: con una velocidad de 10 PDC de 0.83 m/s en comparación a 1.10 m/s para los de controles. Se observó una relación inconsistente entre las puntuaciones de calidad de vida y mobilidad específica y los resultados de las pruebas cronometras para los de controles (correlación 10 PDC= -0.06, 95%*CI* = (-0.41, 0.30)), indica que la percepción de la discapacidad es diferente a la medida de discapacidad, mientras que los resultados para los de controles fueron consistentes (correlación 10 PDC = -0.61, 95%*CI*= (-0.79, -0.34)). Los informes GPS indicaron que los casos generalmente recorren distancias más cortas a velocidades más lentas que los de control, cubriendo un área geográfica más reducida (zona mediana por medio de estimadores kernel: casos = 1.25 km^2, controles = 2.10 km^2, $P = 0.16$). Los casos reportaron tener ingresos inferiores a la mitad de los reportados por los de controles cada semana. (casos = \$0.70, controles= \$1.86, $P = 0.064$), con una proporción más reducida de sus ingresos (16% vs 22%, $P = 0{\cdot}461$) destinada a gastos de salud.

Conclusiones: Aquellos afectados por linfedema muestran una clara desventaja a la de sus pares, estos presentan una calidad de vida más baja tal y como lo afirman ambas medidas de movilidad de sujeto y objeto, y sueldos más bajos. Este estudio además indica que las medidas de movilidad objetivas podrían resultar ser un suplemento útil para auto-evaluar los cuestionarios sobre calidad de vida al momento de evaluar el futuro impacto de las intervenciones de manejo de casos de linfedema.

Translated from English version into Spanish by Evelyn Dench, through

TRANSLATORS
WITHOUT BORDERS
