# Supplementary material for: Measuring the physical and economic impact of filarial lymphoedema in Chikwawa district, Malawi: a case-control study
Source: Infect Dis Poverty. 2017 Apr 3;6:28. doi: 10.1186/s40249-017-0241-2 (PMC5376674; doi:10.1186/s40249-017-0241-2)
Supplement: Supplementary file 2 — Case-control Questionnaire. (DOCX 21 kb) [file 40249_2017_241_MOESM2_ESM.docx]

**Supplementary Material for ‘*Measuring the physical and economic impact of filarial lymphoedema in Chikwawa district, Malawi: A case-control study*’**

**Case-Control Questionnaire**

Date: Location: Researcher: .

Case ID number:

Status**:** □ Control □ Case (Dreyer 1-4) □ Case (Dreyer 5-7)

Affected part of body with swelling: □ Breast □ Arm (one) □ Both arms □ Leg (one)

□ Both legs □ Hydrocele (one) □ Both hydroceles

Please answer following questions.

| **About yourself**: Please tick appropriate answer and/or fill in your answer in each question. | |
| --- | --- |
| 1. Sex | □ Male □ Female |
| 2. Date of birth and age | Day ( ) / Month ( )/ Year ( ) Age ( ) |
| 3. What is your current marital status? | □ Single □Married/co-habiting  □ Widowed □ Divorced/separated |
| 4. How many family members do you live with? | Adults ( ) Children ( ) |
| 5. Who usually takes care of you when you are ill? | ( ) |
| 6. How long have you lived in this community? | □ Less than 5 years □ 5-9 years □ 10+ years □ Lifetime |
| 7. Level of education | □No formal education □ Primary □ Secondary  □ Higher education |
| 8. What is your main source of income? | □ Own work □ Family □Savings □Charity □Pension  □ Other ( ) |
| 9. Do you work? If you work, what is your occupation? | □ No □ Yes ( ) |
| 10· How many hours per week do you usually work? | ( hours per week) |
| 11. How much do you usually earn in a week? | ( ) |
| 12. How many days have you been out of work/ unable to work in the last 3 months? | ( days) |
| 13.. How much do you usually spend on health care (including transportation) in a month? Try to estimate this for yourself only, not your entire family |  |

Please tick which statement best describes your general health over the last month.

**(Use the illustration provided as a guide. Do not tick more than one box in each group)**

| **a) Mobility** | Not applicable | No problem | Mild | Moderate | Severe | Most severe |
| --- | --- | --- | --- | --- | --- | --- |
| 1. Did you have any problems sitting or getting out of a chair? |  |  |  |  |  |  |
| 2. Did you have any problems lying down or standing up from the floor? |  |  |  |  |  |  |
| 3. Did you have any problems going up steps/stairs? |  |  |  |  |  |  |
| 4. Could you walk for one hour without a break? |  |  |  |  |  |  |
| 5. Do you have any problems using public transport |  |  |  |  |  |  |

| **b) Self-care** | Not applicable | No problem | Mild | Moderate | Severe | Most severe |
| --- | --- | --- | --- | --- | --- | --- |
| 1. Do you have any problems dressing yourself? |  |  |  |  |  |  |
| 2. Did you have any problems bathing yourself? |  |  |  |  |  |  |
| 3. Did you have any problems washing your own clothes? |  |  |  |  |  |  |
| 4. Did you have any problems using the toilet by yourself? |  |  |  |  |  |  |
| 5. Do you have any problems accessing medical help when you need it? |  |  |  |  |  |  |

| **c) Usual activitie**s | Not applicable | No problem | Mild | Moderate | Severe | Most severe |
| --- | --- | --- | --- | --- | --- | --- |
| 1. Did you have any problems cooking? |  |  |  |  |  |  |
| 2. Did you have any problems cleaning the floors? |  |  |  |  |  |  |
| 3. Did you have any problems shopping? |  |  |  |  |  |  |
| 4. Did you have any problems undertaking your usual employment? |  |  |  |  |  |  |
| 5. Did you have any problems doing your usual leisure activities? |  |  |  |  |  |  |

| **d) Psychological health** | Not applicable | No problem | Mild | Moderate | Severe | Most severe |
| --- | --- | --- | --- | --- | --- | --- |
| 1. Do you feel worried about your health? |  |  |  |  |  |  |
| 2. Do you have any problems with your concentration and memory |  |  |  |  |  |  |
| 3. Do you feel worried about the future? |  |  |  |  |  |  |
| 4. Do you feel neglected by friends and family members? |  |  |  |  |  |  |
| 5. Do you feel lonely? |  |  |  |  |  |  |

| **e) Social participation** | Not applicable | No problem | Mild | Moderate | Severe | Most severe |
| --- | --- | --- | --- | --- | --- | --- |
| 1. Did you have problem in moving around freely without feeling self-conscious? |  |  |  |  |  |  |
| 2. Did you have problem in approaching people in the community? |  |  |  |  |  |  |
| 3. Did you have problem joining in regular social activities? (meeting friends/family, playing sports etc,) |  |  |  |  |  |  |
| 4. Do you have any problems attending church/taking part in religious activities? |  |  |  |  |  |  |
| 5. How much of a problem is your overall health? |  |  |  |  |  |  |

The overall disability score = Total score / ( 4 × number of answered questions) × 100

The domain score = Total domain score / ( 4 × number of answered questions) × 100

Notes

…………………………………………………………………………………………………………………………….……………………………………………………………………………………………………………………………………………………………………………………………………………………………………………………………………………………………………………………

| **Pictorial scale for Case-Control Questionnaire**  Please choose the column that best describes the level of difficult you have with the specified task | | | | | | | | |  |
| --- | --- | --- | --- | --- | --- | --- | --- | --- | --- |
|  |  |  |  |  |  |  |  |  |  |
|  |  |  |  |  |  |  |  |  |  |
|  |  |  |  |  |  |  |  |  |  |
|  |  |  |  |  |  |  |  |  |  |
|  |  |  |  |  |  |  |  |  |  |
|  |  |  |  |  |  |  |  |  |  |
| 1 |  | 2 |  | 3 |  | 4 |  |  |  |
|  |  |  |  |  |  |  |  |  |  |

**Comparison of timed test results**

| **Timed tests** |  |  | **Mean** | **SD** | **95% CI**  **of difference** | **p-value** |
| --- | --- | --- | --- | --- | --- | --- |
| **10m walking test (m/s)** | Males | Cases | 0.858 | 0.304 | 0.243, 0.652 | 0.001 |
|  |  | Controls | 1.305 | 0.2433 |  |  |
|  |  |  |  |  |  |  |
|  | Females | Cases | 0.818 | 0.184 | 0.084, 0.345 | 0.003 |
|  |  | Controls | 1.031 | 0.295 |  |  |
|  |  |  |  |  |  |  |
| **Timed up and go (secs)** | Males | Cases | 15.109 | 6.600 | -8.868 -2.002 | 0.007 |
|  |  | Controls | 9.674 | 2.982 |  |  |
|  |  |  |  |  |  |  |
|  | Females | Cases | 14.564 | 5.227 | -5.073 -0.637 | 0.014 |
|  |  | Controls | 11.765 | 3.732 |  |  |
